# Adult Onset Still's Disease: A Review on Diagnostic Workup and Treatment Options

**DOI:** 10.1155/2016/6502373

**Published:** 2016-03-03

**Authors:** Rajesh Gopalarathinam, Eric Orlowsky, Ramesh Kesavalu, Sreeteja Yelaminchili

**Affiliations:** ^1^Department of Internal Medicine, Allegheny General Hospital, 320 E. North Avenue, Pittsburgh, PA 15212, USA; ^2^Department of Rheumatology, Allegheny General Hospital, 320 E. North Avenue, Pittsburgh, PA 15212, USA; ^3^Department of Rheumatology, Lakeside Community Healthcare Medical Group, 1500 S. Central Avenue No. 200, Glendale, CA 91204, USA; ^4^Department of Internal Medicine, Capital Health Regional Medical Center, 750 Brunswick Avenue, Trenton, NJ 08638, USA

## Abstract

Adult onset Still's disease (AOSD) is a rare systemic inflammatory disease of unknown etiology and pathogenesis that presents in 5 to 10% of patients as fever of unknown origin (FUO) accompanied by systemic manifestations. We report an interesting case of a 33-year-old African-American male who presented with one-month duration of FUO along with skin rash, sore throat, and arthralgia. After extensive workup, potential differential diagnoses were ruled out and the patient was diagnosed with AOSD based on the Yamaguchi criteria. The case history, incidence, pathogenesis, clinical manifestations, differential diagnoses, diagnostic workup, treatment modalities, and prognosis of AOSD are discussed in this case report.

## 1. Introduction

AOSD is characterized by the classic triad of persistent high spiking fever, arthralgia, and salmon colored skin rash. However, diagnosing AOSD is often difficult due to the presence of several nonspecific symptoms and the absence of characteristic serological biomarkers. AOSD is typically considered as a diagnosis of exclusion and a definitive diagnosis should be made based on the Yamaguchi or Fautrel criteria only after excluding infectious, malignant, and other connective tissue diseases. Timely diagnosis and treatment of the disease with corticosteroids followed by maintenance therapy with disease modifying antirheumatic drugs (DMARDs) or biologic drugs such as tumor necrosis factor- (TNF-) alpha agents or interleukin (IL-1) antagonists can prevent complications and lead to a favorable prognosis.

## 2. Case Presentation

A 33-year-old African-American male presented to the emergency department with a 4-week history of high-grade fever, sore throat, and dry cough. His fever was accompanied by nonpruritic macular skin rash on his trunk, arthralgia of bilateral ankles and knees, myalgia, and night sweats. He denied the presence of joint stiffness in the mornings, blurry vision, eye pain, oral ulcers, headache, back pain, burning urination, recent travel, sick contacts, decreased appetite, or weight loss. The rest of the review of the systems was negative. He had no significant past medical history, was not taking any medications previously, and had no significant family history. He had no known allergies. On physical exam, patient was febrile with a temperature of 39 degrees Celsius, tachycardic with a heart rate of 110 beats per minute, tachypneic with a respiratory rate of 22 breaths per minute and a blood pressure of 130/80 mmHg. Patient had a maculopapular skin rash on the chest, abdomen, and back. He also had inflamed throat without any exudates or cervical lymphadenopathy. Musculoskeletal exam showed minimal tenderness in bilateral ankles and knees with normal active and passive range of motion. There were no signs of active synovitis in any of his joints. Abdomen exam showed no organomegaly. Cardiovascular, respiratory, and neurological exam were unremarkable. He was admitted to the hospital and his initial workup revealed elevated acute phase reactants (Erythrocyte sedimentation rate (ESR): 47 mm/hr, C-reactive protein (CRP): 80 mg/L, and ferritin > 2000 *μ*g/L), mildly elevated liver function tests (aspartate transaminase: 75, alanine transaminase: 90, and alkaline phosphatase: 100), normocytic anemia with normal white cell count with neutrophilic predominance (80%), and normal platelet count. Rapid strep throat test, HIV, hepatitis panel, blood cultures, throat cultures, and urine analysis were all negative. Anti-nuclear antibody (ANA), rheumatoid factor (RF), and cyclic citrullinated peptide (Anti-CCP) antibody were negative too. Chest X-ray showed no acute infiltrates. Subsequently, the patient was referred to an infectious disease specialist for his fever of unknown origin (FUO). The patient received a course of antibiotic (levofloxacin) during hospitalization which did not resolve his symptoms. A few days following admission, the patient experienced nausea, vomiting, and left upper quadrant abdominal pain without diarrhea. An abdomen and pelvis CT was done which was normal except for a borderline splenomegaly. An extensive ID workup was ordered which included fungal serologies and gallium scan, all of which turned out to be normal. Hematology was consulted for the patient's FUO and normocytic anemia. Hematological conditions such as leukemias, lymphomas, and hemophagocytic lymphohistiocytosis were ruled out due to the absence of physical and laboratory findings. A 2D echo, which was ordered to rule out endocarditis, came back as a normal study. As several investigations including serum protein electrophoresis and urine protein electrophoresis were nondiagnostic for the patient's FUO and anemia, a bone marrow biopsy with flow cytometry was finally ordered. The bone marrow study showed mildly hypercellular pattern with adequate storage of iron and no evidence of fibrosis or lymphoproliferative disorder. Rheumatology was consulted for his FUO in relation to arthralgia and myalgia. An anti-neutrophil cytoplasmic antibody (ANCA) panel, Lyme disease serology, and HLA-B27 tests were done and the results came back negative. After ruling out sickle cell anemia, tuberculosis, and other infectious causes, a diagnosis of adult onset Still's disease was made based on the Yamaguchi criteria. Subsequently, the patient was started initially on high dose prednisone 60 mg PO daily and then methotrexate 10 mg once weekly was added for steroid sparing effect. Since then, the patient improved and the intensity of his fever, skin rash, and ankle and knee pain came down significantly. The patient was discharged home in a stable condition with outpatient followup instructions. During the next clinic visit in 2 weeks, his fever, rash, and arthralgia subsided completely and his steroid dose was tapered appropriately. He did clinically well on methotrexate 20 mg once weekly.

## 3. Discussion

AOSD is a rare systemic inflammatory disease of unknown etiology. It was initially described by Bywaters in 1971 as a distinct clinical entity in adults that is quite similar to the one observed in children known as systemic juvenile idiopathic arthritis (sJIA) [1]. It has an estimated prevalence of 1.5 cases per 100,000–1000,000 people. It has been described all over the world and has a bimodal age distribution with 2 peaks, the first peak affecting people within 15–25 years of age and the second peak affecting people within 36–46 years of age [2]. Although it usually affects the younger adult population, it can also affect elderly people [3]. The disease affects predominantly females as compared to males [4].

The exact pathogenesis of AOSD is unknown. Several factors such as genetics, infectious (bacterial and viral) agents, and environmental factors have been thought to play a causative role [5–8]. But there are no strong evidences to suggest their causal relationship with the disease. An important step in the pathogenesis of AOSD is interleukin-18 (IL-18) mediated macrophage and neutrophil activation (evidenced by upregulation of CD 64 in patients with active disease) [9]. The pathogenesis of AOSD involves the interplay of numerous activated cytokines. Serum levels of tumor necrosis factor- (TNF-) alpha, IL-1, IL-6, IL-18, Interferon gamma IFN-*γ*, IL-8, and Soluble interleukin-2 receptor SIL-2R have been found to be elevated in patients with active AOSD [10]. Among the various cytokines, interleukin 1*β* (IL-1*β*) seems to be emerging as a key regulator in the pathogenesis of AOSD. It is involved in proliferation of neutrophils and diapedesis. Several molecular studies have also shown that elevated levels of IL-1*β* are associated with active and severe disease [11]. It has also been demonstrated in several studies that high levels of IL-18 in AOSD patients are a predictor of liver dysfunction [12].

**Figure 1:**
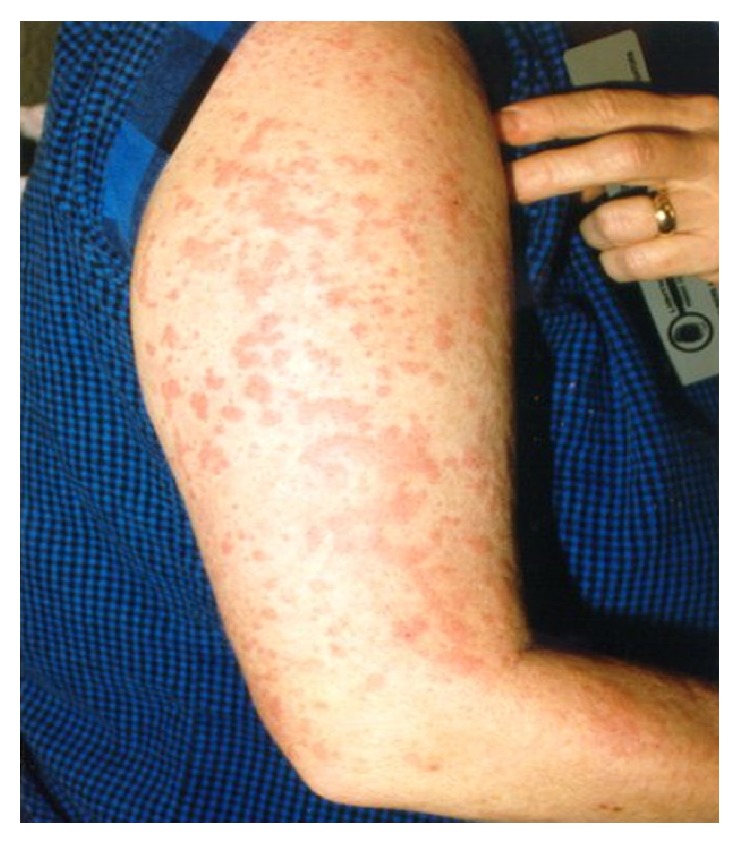
Characteristic salmon colored, nonpruritic, and maculopapular rash of Still's disease (source: http://www.stillsdisease.org/).

**Figure 2:**
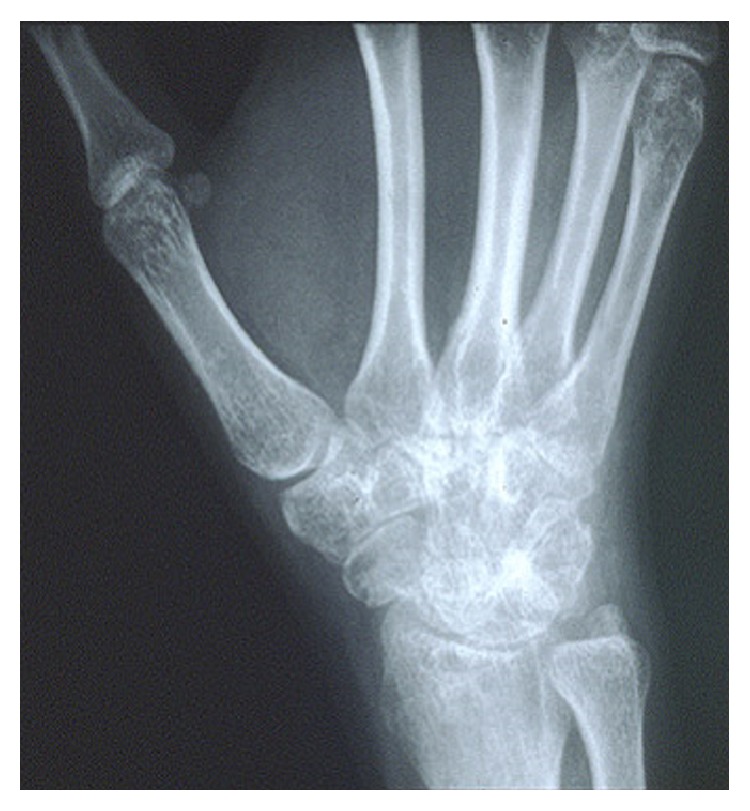
Xray of the right wrist showing diffuse narrowing of the radiocarpal and carpometacarpal joints of the wrist which is a characteristic finding in Still's disease. (Source: http://www.hopkinsarthritis.org/)

Clinically, the most classic manifestations of AOSD are fever, rash, sore throat, and arthralgia with fever and arthralgia being the most common among them [13]. The fever is usually a high spiking quotidian fever (≥39°C) that occurs in the evening with return of normal temperature the next day morning. The fever is often accompanied by other symptoms or could present as FUO alone [14]. In our patient, his high-grade fever occurred mostly during the evenings and was accompanied by pharyngitis and maculopapular skin rash. Nonsuppurative pharyngitis is one of the common earlier findings in AOSD and can either precede the development of fever or can occur along with other symptoms. The pharyngitis in AOSD patients is proposed to be from underlying cricothyroid perichondritis [15]. The characteristic rash in Still's disease is a transient, nonpruritic, salmon colored, macular, or maculopapular lesion often observed during febrile episodes (see Figure 1) [16]. The most common locations of the rash include the trunk and proximal extremities. In one-third of patients, the rash maybe mildly pruritic and can develop at sites of cutaneous injury due to pressure or trauma, which is referred to as Koebner's phenomenon [17]. Skin biopsy of the rash shows a mild nonspecific perivascular inflammation. Immunofluorescence of skin biopsy shows perivascular deposition of C3 protein [17]. Another common finding in many AOSD patients is an exaggerated urticarial response to cutaneous stimuli (i.e., the scratch test) which is referred to as dermatographism [18]. Intense arthralgia is ubiquitously seen in all AOSD patients. The most commonly involved joints are the knees, wrists, ankles, and elbows [6]. AOSD often involves the distal interphalangeal joints of the hand, which are commonly spared in inflammatory joint disease of the young adults (e.g., SLE and rheumatoid arthritis) with the exception of psoriatic arthritis. Narrowing of the carpometacarpal spaces of the hands is also found to be specific for AOSD (See Figure 2) [19]. Joint fluid analysis often shows marked neutrophilic leukocytosis [14]. Our patient had involvement of bilateral ankles and knees with sparing of other joints including hands, wrists, and elbows. Myalgia, which is usually generalized and severe, is a frequently encountered symptom during febrile episodes [6]. In all AOSD patients, throat cultures and viral serologies are negative and, therefore, antibiotic therapy is ineffective [2, 20]. Lymphadenopathy develops in 44–90% of AOSD patients [5, 21] and may raise the suspicion of lymphoma initially [22]. Hepatosplenomegaly can be a common manifestation in the early phase of the disease. Less commonly, pericarditis or pleuritis may occur. Macrophage activation syndrome (MAS) is a life threatening complication seen in AOSD as well as systemic onset juvenile idiopathic arthritis (sJIA). Its pathogenesis is not clearly understood but involves cytokine induced hyperproliferation of activated CD8+ T-lymphocytes and macrophages in the reticuloendothelial system [23]. It should be suspected in ASOD patients who present with fever, hepatosplenomegaly, lymphadenopathy, pancytopenia, transaminitis, coagulopathy, and CNS/pulmonary/renal involvement. Bone marrow aspiration and biopsy which show hemophagocytosis help in establishing the diagnosis of MAS [23]. Another clinical condition that closely mimics macrophage activation syndrome is the reactive hemophagocytic system (RHS) [24].

The laboratory tests in AOSD show features suggestive of an inflammatory process. The most common laboratory abnormalities include

- elevated erythrocyte sedimentation rate (ESR),
- leukocytosis (in most cases within 15,000–30,000, mainly neutrophils),
- thrombocytopenia > 400,000,
- elevated ferritin levels.

Elevated ferritin level is a nonspecific but common finding and a helpful feature for diagnosing AOSD [25]. The ferritin levels are often higher (>2000 mg/mL) than the levels found in other autoimmune or inflammatory disease and are likely secondary to cytokine secretion induced by the reticuloendothelial system [25]. Among the several isoforms of ferritin that have been described, one that deserves mention is the glycosylated ferritin which is typically decreased in AOSD patients. Studies have shown that a combination of glycosylated ferritin fraction < 20% and ferritin level above the upper limit of the normal range improved the sensitivity and specificity to 70.5% and 83%, respectively, as compared with using elevated ferritin levels alone [25, 26]. Our patient had a glycosylated ferritin 18%. Although hyperferritinemia is often helpful in diagnosing AOSD, normal levels of serum ferritin should not rule out the diagnosis of AOSD [27]. Glycosylated ferritin should not be used to monitor the disease activity or response to treatment, because it remains low for many months even after the disease goes into remission. Other less common laboratory findings in AOSD include serum albumin < 3.5 gms/dL, anemia of chronic disease, and elevated hepatic transaminase levels [5]. Rheumatoid factor and antinuclear antibody tests are usually negative [5, 6]. Synovial and serosal fluids are inflammatory type with neutrophilic predominance [14]. The radiographic results are generally normal in the early phase of the disease and maybe helpful in the late and chronic phase with worsening erosions and joint space narrowing (carpometacarpal joints are more commonly involved than the tarsometatarsal joints) [19, 28].

Diagnosing AOSD is often difficult due to the presence of several nonspecific symptoms and the absence of characteristic serological biomarkers. The Yamaguchi criteria are the most widely cited criteria and are shown to be the most sensitive ones (93%) [29]. The major and the minor criteria of the Yamaguchi criteria are shown below.

Major criteria are as follows:

- Fever of at least 39°C for at least a week.
- Arthralgia or arthritis for at least 2 weeks.
- Nonpruritic salmon colored rash on trunk/extremities.
- Granulocytic leukocytosis (10,000/microL or greater).

Minor criteria are as follows:

- Sore throat.
- Lymphadenopathy.
- Hepatomegaly or splenomegaly.
- Abnormal liver function tests.
- Negative tests for RF and ANA.

Diagnosis requires at least 5 features, with at least 2 of these being major diagnostic criteria. Our patient had all 4 of the major criteria and all of the minor criteria except lymphadenopathy.

In 2002, Fautrel et al. proposed a new criterion which contained 2 new markers: serum ferritin and glycosylated ferritin. The sensitivity and specificity of Fautrel criteria were 80.6% and 98.5%, respectively [30]. The Fautrel diagnostic criteria for AOSD are shown below.

Major criteria are as follows:

- Spiking fever ≥ 39°C.
- Arthralgia.
- Transient erythema.
- Pharyngitis.
- Neutrophilic polymorphonuclear count ≥ 80%.
- Glycosylated ferritin fraction ≤ 20%.

Minor criteria are as follows:

- Typical Still's rash.
- Leukocytosis (10,000/mm^3^).

Diagnosis of AOSD by Fautrel criteria requires 4 or more major criteria or 3 major and 2 minor criteria. Our patient had all 5 major criteria and 2 minor criteria.

AOSD is primarily a diagnosis of exclusion which should be considered only after excluding several other differential diagnoses [2, 5, 14, 31]. Among many differentials, the major diseases that should be excluded are as follows:

1. Infections: viral infections (Rubella, Epstein-Barr virus, Cytomegalovirus, HIV, hepatitis B and C, coxsackie, and Parvovirus), infective endocarditis, Lyme disease, and tuberculosis.
2. Granulomatous diseases: Sarcoidosis, Crohn's disease, and idiopathic granulomatous hepatitis.
3. Malignancy: leukemias and lymphomas.
4. Connective tissue diseases: systemic lupus erythematosus (SLE), mixed connective tissue disease (MCTD), polyarteritis nodosa (PAN), Wegener's granulomatosis, and Takayasu's arteritis.

Blood cultures and serological tests can be useful to rule out infections. Malignant disorders can be differentiated from AOSD by their hematological profile, but sometimes bone marrow and/or lymph node biopsy may be needed [32]. The familial Mediterranean fever (FMF) and TNF receptor associated periodic syndrome (TRAPS) are two syndromes that fall under the category of periodic fever syndromes that closely resemble the clinical picture of AOSD [33]. Patients with FMF often present with acute, self-limiting episodes of fever that last only for 1–3 days. Unlike AOSD, the fever in FMF does not have the typical quotidian pattern and is accompanied by serositis or acute synovitis of the hip, knee, or ankle. The fever can be accompanied by erysipelas like skin rash. FMF usually starts in childhood. The familial distribution of the disease together with the distinct clinical characteristics and response to Colchicine can help the physician to get the correct diagnosis. Genetic analysis for the MEFV gene is also used to verify the diagnosis in suspected cases. With TRAPS, the fever lasts longer (≥3 to 4 weeks on average) and is associated with ocular manifestations and a characteristic centrifugal erythematous patch. Both FMF and TRAPS often begin during childhood and have a strong familial distribution.

The disease pattern of patients with AOSD can be divided into 3 distinct types [6, 14, 32]:

1. Monocyclic or self-limiting pattern, which has a single episode of systemic disease of variable duration followed by complete remission.
2. Polycyclic or intermittent pattern, where 2 or more episodes of systemic disease are separated by symptom-free remission period lasting for a minimum of 2 months.
3. Chronic articular pattern, which is characterized by the severe articular manifestations causing joint destruction.

In several studies, a poor prognosis is associated in AOSD patients with polyarticular onset, proximal joint arthritis, prior episode in childhood, and requirement of systemic steroids for more than 2 years [6, 34]. In contrast, patients with monocyclic or polycyclic systemic disease, no arthritis at presentation, or an oligoarticular onset and course showed better functional status.

Corticosteroids remain the first-line treatment for AOSD, regardless of the clinical presentation. Usually, prednisolone is preferred among steroids. Steroids control symptoms in about 60% of AOSD patients. Intra-articular steroid injection can be used in the treatment of chronic articular pattern of AOSD [35]. Disease modifying antirheumatic drugs (DMARDs), such as methotrexate (MTX), azathioprine, cyclosporine, and cyclophosphamide, are often used for maintenance therapy of the disease [4, 32]. Sulfasalazine causes severe adverse reactions in AOSD (ranging from gastrointestinal symptoms such as abdominal pain, nausea, and vomiting to severe myelosuppression) and should be avoided [36]. MTX is often used due to its steroid sparing effect and its potential to prevent worsening of arthritis [37]. Cyclosporine can be used for AOSD patients presenting with macrophage activation syndrome (MAS). Since several cytokines such as TNF-alpha, IL-1, and IL-6 are implicated in the pathogenesis of AOSD, biologic agents can be used in refractory AOSD patients [38, 39]. While several studies show the efficacy of anti-TNF agents (infliximab, etanercept, and adalimumab) in refractory AOSD [40–43], IL-1 inhibition is considered the mainstay of treatment for refractory AOSD leading to significant improvement in both clinical and laboratory terms [44, 45]. Three IL-1 antagonists are presently available, that is, a recombinant antagonist of the IL-1 receptor (IL-1Ra, anakinra), a human monoclonal antibody directed against IL-1*β* (canakinumab), and a soluble IL-1 trap fusion protein (rilonacept). Among the three IL-1 antagonists, anakinra has been used more frequently. Anakinra is particularly efficient in the rapid relief of systemic symptoms. Its effect on articular symptoms is less frequently reported. It is administered subcutaneously once daily, due to its short half-life. In the event of an insufficient response to anakinra, rilonacept and canakinumab can be considered because they have longer half-lives and can be administered every week or every 8 weeks, respectively [46, 47]. Tocilizumab is another monoclonal antibody directed against the IL-6 receptor that has been shown to induce remission in patients with AOSD refractory to standard treatment from several case studies. It is also used in the treatment of systemic onset juvenile idiopathic arthritis (sJIA) and is usually administered as a monthly intravenous infusion or a subcutaneous injection every 1-2 weeks. It has a marked corticosteroid-sparing effect and a good tolerance profile [48, 49]. Plasma exchange and intravenous immunoglobulins are other treatment options in refractory AOSD patients [50, 51].

In conclusion, over the past four decades, AOSD still remains as a diagnostic dilemma for physicians as it presents with a combination of nonspecific symptoms that can be caused by a wide variety of diseases. However, the key point to remember is that, for patients who present with prolonged and unexplained fever combined with musculoskeletal symptoms and macular rash, the differential diagnoses should include AOSD. Timely diagnosis and treatment of the disease can prevent complications and lead to a favorable prognosis with improved quality of life.
